# Effects of rifle carriage on performance, physiological responses, power output and external work in a simulated biathlon competition

**DOI:** 10.1007/s00421-026-06301-x

**Published:** 2026-06-29

**Authors:** M. Jonsson Kårström, M. Horvath, E. P. Andersson, M. S. Laaksonen

**Affiliations:** Swedish Winter Sports Research Centre, Department of Health Sciences, Mid Sweden University, 831 25 Östersund, Sweden

**Keywords:** Cross-country skiing, Elite athletes, Oxygen uptake, Pacing, Skiing speed

## Abstract

**Purpose:**

To investigate the effect of rifle carriage on performance, physiological responses, power output and external work during a simulated biathlon competition in the field.

**Methods:**

Twenty well-trained biathletes (10 women) completed a simulated biathlon competition (women 3 × 1.9 km, men 3 × 2.4 km) twice, one trial while carrying the rifle (WR) and the other without rifle (NR) in a randomized counter-balanced order. Laps were separated by two simulated shootings, lasting 38.9 ± 8.2 s. Trials were separated by 30-min of active recovery. Heart rate and respiratory variables were measured throughout each trial. Furthermore, blood lactate concentration and ratings of perceived exertion were assessed after each lap. Power output and external work were estimated for both trials based on positional data.

**Results:**

Overall roller-skiing speed was lower WR than NR (5.49 ± 0.47 vs. 5.69 ± 0.49 m∙s^−1^, p < 0.001), with largest differences during the uphill sections. No differences in physiological responses were found between WR and NR during laps or course sections (p > 0.05), but oxygen uptake and breathing frequency were higher WR than NR during the second simulated shooting (p < 0.05). Mean power output was slightly lower WR than NR; however, the participants performed more external work WR compared to NR due to the longer duration of the WR trial (both p < 0.05).

**Conclusion:**

Since elevated physiological markers may negatively impact standing shooting performance, biathletes should optimize their pacing strategies to maximize overall performance. In addition, rifle carriage increases external work through a lengthened skiing duration, which should be considered when planning and performing biathlon training.

**Supplementary Information:**

The online version contains supplementary material available at https://doi.org/10.1007/s00421-026-06301-x.

## Introduction

During a biathlon competition, the biathlete performs high-intensity cross-country (XC) skiing using the ski-skating technique at an average intensity corresponding to ~ 90% of the maximal heart rate (HR_max_) (Hoffman and Street 1992) while carrying a rifle (minimum mass 3.5 kg, IBU (2024)). The skiing is interrupted by 2–4 shootings, each lasting approximately 25‒35 s at the highest competition level (Laaksonen et al. 2018b), and generates an additional skiing loop or time penalty for each missed target. Thus, success in biathlon races is a result of optimized skiing and shooting speed and a high shooting accuracy (Laaksonen et al. 2018a).

The biathlon sprint competition is performed with an interval start, and consists of three laps of skiing (each lap is 2.5 km for women, and 3.3 km for men) separated by one prone and one standing shooting (IBU 2024), typically lasting 20‒23 min depending on the course profile and snow conditions (Laaksonen et al. 2018b). In biathlon sprint events, skiing speed is the dominant determinant distinguishing top 10 finishers from those finishing 20‒30 in World Cup competitions (Luchsinger et al. 2018). Nevertheless, a high level of shooting accuracy remains crucial to be in contention for the top positions (Björklund 2018; Björklund et al. 2021; Björklund and Laaksonen 2022).

From a physiological perspective, skiing with a rifle (WR) increases heart rate (HR), oxygen uptake (VO_2_), minute ventilation (VE), metabolic energy expenditure, respiratory exchange ratio (RER), and blood lactate concentration (BLa) compared to skiing at the same submaximal speeds with no rifle (NR) (Jonsson Kårström et al. 2019; Rundell and Szmedra 1998; Stöggl et al. 2015). Nevertheless, since the speeds during these laboratory studies were fixed during the submaximal workloads, the physiological differences between WR and NR can partly be explained by the increased mechanical work performed when roller-skiing WR (due to the mass of the rifle). However, no studies have examined the physiological effects of rifle carriage during self-paced biathlon skiing in an ecological field setting.

The maximal power and capacity of the aerobic energy system is of great importance for skiing speed, with both maximal/peak VO_2_ (VO_2max_/VO_2peak_) and performance VO_2_, and even more importantly, the speed at the second lactate threshold (4 mmol∙L^−1^), which have been shown to determine biathlon and XC skiing performance (Carlsson et al. 2012; Jonsson Kårström et al. 2019; Laaksonen et al. 2020; Sandbakk et al. 2011, 2016; Talsnes et al. 2021; Tønnessen et al. 2015). Biathlon and XC skiing competitions are performed on varied terrain, including undulating uphill, flat, and downhill sections, resulting in highly intermittent physiological characteristics (Falk Neto et al. 2024). In general, approximately 50% of the total competition time in XC skiing is spent in uphill sections, with the remaining time spent on flat (35%) and downhill (15%) sections (Losnegard 2019). Due to the changes in course characteristics, large fluctuations in propulsive power and metabolic energy expenditure occur. XC skiers have been found to frequently perform at work rates corresponding to ~ 110–160% of their VO_2peak_ (depending on the duration of the uphill section) during simulated roller-skiing competitions (Andersson et al. 2019; Gløersen et al. 2020; Karlsson et al. 2018; Losnegard 2019). This indicates that both the capacity and the restoration rate of anaerobic energy systems are important performance factors in XC skiing and biathlon competitions (Andersson et al. 2016; Gløersen et al. 2020).

When training, biathletes ski both WR and NR. Skiing WR increases physiological responses compared to NR when skiing at the same speed (Jonsson Kårström et al. 2019; Rundell and Szmedra 1998; Stöggl et al. 2015), which suggests that skiing speed is not a suitable metric for evaluating training intensity between these training conditions. Furthermore, skiing speed is affected by environmental factors (e.g., terrain, snow conditions, and temperature), and the used material (e.g., waxing and structure of the skis). During low-intensity biathlon training (including shooting, i.e., WR) biathletes showed lower external intensity (average net force), but similar internal intensity (HR) and duration compared to skiing without shooting (i.e., NR), with no differences for high-intensity training (Kårström et al. 2024). Vertical distance of the upper-body has been shown to decrease during WR compared to NR skiing (Jonsson Kårström et al. 2023), besides more work being performed by the lower-body during WR (Stöggl et al. 2015), which may affect the measurement of average net force, since the sensor often is placed at the upper-body (as in the study by (Kårström et al. 2024)). In addition, in the study by Kårström et al., rifle mass was omitted from the calculation of the average net force. However, since power output can account for the effect of the increased system mass (i.e., the summed mass of the biathlete and the rifle), it might be a suitable metric for accurately monitoring external intensity during both WR and NR skiing as suggested by Jonsson Kårström et al. (2019).

Altogether, rifle carriage increases the physiological demand during submaximal workloads compared to skiing NR at the same speeds and inclines (Jonsson Kårström et al. 2019; Rundell and Szmedra 1998; Stöggl et al. 2015). However, the physiological response to rifle carriage during a self-paced simulated competition (i.e., including maximal effort) on varying terrain is unclear. Therefore, the present study aimed to investigate the effect of rifle carriage on performance, physiological responses, power output and external work during a simulated biathlon competition in the field.

## Methods

### Participants

Twenty biathletes (10 women and 10 men) volunteered to participate in the study (Table 1). All participants trained and competed regularly in biathlon and represented tier 4 and tier 3 athletes (elite/international level and highly trained/national level) according to the participant classification framework by McKay et al. (2022). Participants received both written and verbal information before testing, regarding the study’s purpose, potential benefits and risks, and their right to withdraw at any time without consequences. Written informed consent was obtained from all participants. The study was pre-approved by the Swedish Ethical Review Authority (Ref 2023–04516-01).

**Table 1:** Participant characteristics (n = 20, including 10 female and 10 male biathletes)

Age (years)	21.7 ± 2.6
Body height (cm)	177 ± 10
Body mass (kg)	71.7 ± 10.3
Rifle mass (kg)	3.88 ± 0.25
Rifle mass (% of body mass)	5.5 ± 0.8
Annual training volume (hours)	480 ± 123

### Study design

**Fig. 1:**
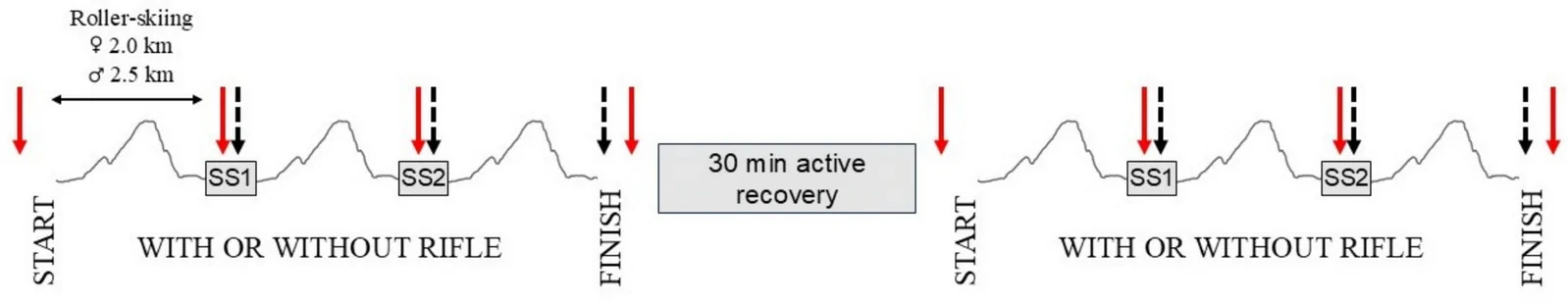
Schematic overview of the testing procedure. Small grey boxes symbolize the simulated shootings performed in the prone (SS1) and standing (SS2) positions. The solid red arrows indicate blood lactate sampling, whereas dashed black arrows denote the ratings of perceived exertion (RPE). The two trials (with and without a rifle) were separated by a 30-min active recovery period at self-selected intensity

Each participant completed two simulated sprint competitions (3 × 1.9 km for women, 3 × 2.4 km for men), one with the rifle (i.e., WR) and one without (i.e., NR), in a randomized counterbalanced order. Between the two sprint trials, participants had 30 min of active recovery at a self-selected intensity (Fig. 1). All tests were performed at the Swedish National Biathlon Stadium in Östersund, and each participant was tested on a single day. The data collection was completed between June and September. The weather conditions were fairly stable during a single testing session but varied between testing days (ambient temperature varied between 11 and 24 °C, mean temperature 15 ± 3 °C). Tests were performed as roller-skiing and all athletes used the same pair of roller-skis (Marwe (wheels: type 6); Marwe Oy, Hyvinkää, Finland) as well as their own ski boots, poles and biathlon rifle.

### Testing procedure and measurements

At arrival to the stadium, the participant rested in a seated position for 10 min before a capillary blood sample was taken from the fingertip for measurements of BLa (Biosen S_Line, EKF diagnostics, Magdeburg, Germany). Body height, body mass and rifle mass were measured, after which participants were equipped with a chest-strap HR monitor (HRM-Pro Plus, Garmin Ltd, Kansas City, Missouri, USA) and a wrist-worn standalone global navigation satellite system (GNSS) receiver with integrated inertial measurement units (IMUs) (Garmin Forerunner 955; Garmin Ltd, Kansas City, Missouri, USA). Prior to the simulated sprint trials, the participants performed a 30-min warm-up on roller-skis. The participants were instructed to fully complete their respective sprint course at least once and to include one bout of exercise at competition intensity lasting 2–3 min during the second half of the warm-up. The remaining parts of the warm-up were performed according to their own preference. Following the warm-up, a capillary blood sample was taken for the measurement of Bla, after which the participant was equipped with a portable breath-by-breath indirect calorimetry system (MetaMax3B_R2; Cortex Biophysik GmbH, Leipzig, Germany) and their biathlon rifle, depending on the order of trials.

After laps 1 and 2 during the trial, the participant stopped for a simulated shooting in either the prone or the standing position, where BLa and a Rating of Perceived Exertion scores (RPE, scale 6–20) (Borg 1970) were collected (Fig. 1). The duration of each stop depended on the time needed for the measurements, but typically lasted between 30 and 45 s, which approximates ordinary shooting times at this performance level. Participants were instructed to pace their skiing during both WR and NR conditions, as they would during a real sprint race involving shooting. Upon completion of each trial, RPE scores were collected immediately post exercise, and Bla was collected after two minutes. Subsequently, the participants performed a 30-min active recovery (roller-skiing) at a self-selected intensity, a duration shown to allow sufficient BLa recovery (Staunton et al. 2022; Vesterinen et al. 2009), before repeating the simulated sprint trial under the remaining condition (i.e., WR or NR).

Respiratory variables (i.e., VO_2_, carbon dioxide production (CO_2_), RER, VE and breathing frequency (BF)), HR and the athletes’ position on the track were continuously recorded throughout both trials. Values of HR are presented relative to each participant’s self-reported HR_max_. Before each trial, the portable breath-by-breath calorimetry system was calibrated following the manufacturer’s instructions using a 3-L syringe and a calibration gas mixture with known gas concentrations of 15.0% O_2_ and 5.0% CO_2_.

### Course characteristics

**Fig. 2:**
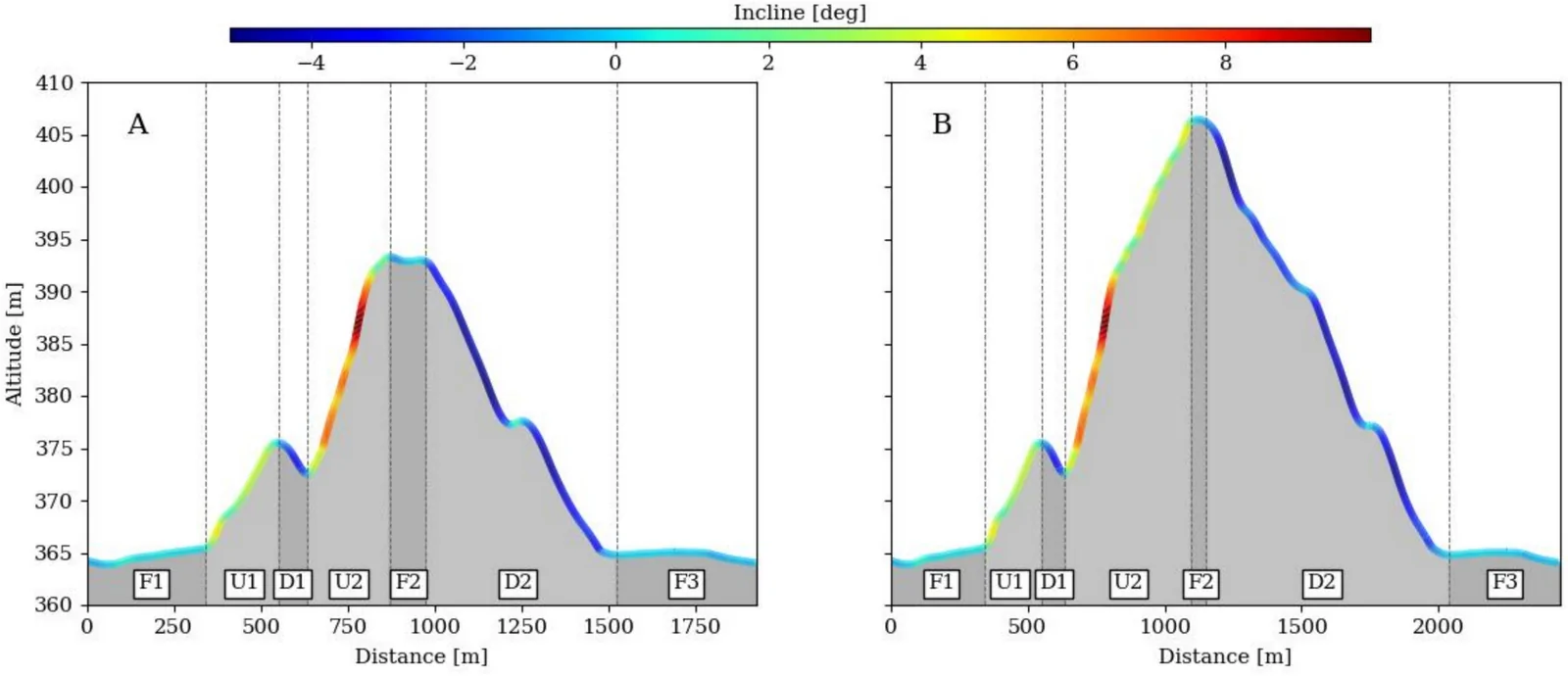
Two-dimensional representation of the course profiles with marked sections for the 1.9-km course completed by the female (**A**), and the 2.4-km course completed by the male participants (**B**). The hue (i.e., color shade) of the course profiles indicates the gradient. *F * flat section, *U* uphill section, *D* downhill section

The female participants skied three laps on a 1.9 km course, while the male biathletes completed 3 × 2.4 km. The total elevation gain per lap was 35 m for the female course, and 48 m for the male course, with the longest individual climbs measuring 21 m and 34 m of elevation gain, respectively. Based on the courses’ terrain characteristics, each lap was divided into seven distinct sections for both sexes: two uphill (U1 and U2), three flat (F1, F2, and F3), and two downhill sections (D1 and D2) (Fig. 2).

### Missing data

Due to blockage of the gas exchange analyzer’s sampling line, values for VO_2_ and RER were not recorded and available for 1–4 participants, depending on course section. One participant had no recorded values for VO₂ and RER for one of the races. Another participant had no recorded values for the two final sections of lap 2 or for the entire third lap, while two participants had no recorded values for the two final sections of the final lap. Additionally, one participant skied a slightly different course than the other biathletes, which affected time and speed in sections D1 and U2. Consequently, time and speed data for these sections for that participant were excluded from the analysis. However, physiological data from this participant were retained, as the same course was skied during all three laps in both trials, and this deviation was therefore considered to have had a limited effect on physiological values.

### Data processing

Positional coordinates were sampled every second and filtered by manufacturer-defined algorithms inherent to the used GNSS receiver, before being converted to east-north coordinates in Python (v.3.12, Python Software Foundation, Wilmington, DE, USA). Based on frequency spectrum analysis, it was concluded that these proprietary filters have sufficiently removed noise from the data.

The converted coordinates were interpolated to the mean measured lap distance (i.e., 1928 m for the female, and 2444 m for the male course) using cubic splines (McKinley and Levine 1998). This process yielded datasets of the same length, resampled to each meter along the measured trajectories. Based on previously published studies, a mapping trajectory neglecting the athletes’ lateral displacement relative to the roller-ski course was created (Gløersen et al. 2018a, 2018b). This trajectory was generated by averaging interpolated laps based on displacement relative to the start position. Finally, the interpolated individual positional datasets were mapped onto their respective mapping trajectories by identifying the nearest trajectory point to each measured position, thereby enabling accurate between-athlete comparisons of speed and position along the course. The derived instantaneous speed signal was smoothed using a 30-m (i.e., ~ 3‒10 s) bidirectional rolling average filter.

When generating altitude profiles for common sections of the female and male courses, the data used for constructing mapping trajectories were merged. Incline was calculated at each meter of the courses, thus generating two respective “reference” incline datasets, which were used during power output and external work calculations later.

Breath-by-breath gas exchange data were linearly interpolated to 1 Hz. According to the guidelines provided by Robergs et al. (2010) and Nolte et al. (2023), the interpolated datasets were post-processed using a bidirectional moving mean filter with a window size of 30 s. Subsequently, the gas exchange and HR data were synchronized with the skier’s position on the reference trajectory, thereby creating two datasets of the same length, enabling accurate comparisons in terms of physiological variables across all sections of the courses. Maximal respiratory values were obtained as the highest processed datapoints (Robergs et al. 2010).

### Work and power calculations

Sub-technique selection throughout the trials was assessed using a portable three-dimensional motion analysis system (MyoMotion, Noraxon Inc., Scottsdale, AZ, USA) comprising twelve IMUs placed on anatomical landmarks, according to the procedure described in previous studies (Jonsson Kårström et al. 2022; Staunton et al. 2022). The system was calibrated before each trial according to the manufacturer’s guidelines. Body kinematics were recorded at a 100 Hz sampling rate (sensor measurement limits: accelerometer: ± 16 g; gyroscope: ± 2000°·s − 1; magnetometer: ± 1.9 gauss). Data were processed by a trained researcher, who classified skating XC skiing sub-techniques as Gear 2, Gear 3, and Gear 4 (for descriptions of sub-techniques, see e.g., Nilsson et al. (2004)). When none of these sub-techniques could be identified based on body kinematics, the movement was classified as no propulsion (i.e., tuck position).

Based on the assumption that the biathlete-rifle system can be described as a point mass moving along the roller-ski course, the average propulsive power output was estimated for each section and lap of the trials using a power-balance approach. This approach accounted for propulsive, gravitational, frictional, and drag forces (Ingen Schenau and Cavanagh 1990; Moxnes and Hausken 2008) as:

1 $$m_{sys} v\frac{dv}{{dt}} = P - m_{sys} g\left( {\sin \alpha + \mu_{r} \cos \alpha } \right)v - 0.5\rho C_{d} Av^{3} ,$$

where the left-hand side represents the rate of change of the system’s kinetic energy over time, ${\mathrm{m}}_{\mathrm{s}\mathrm{y}\mathrm{s}}$ denotes the system mass, $\mathrm{v}$ is the speed of travel, and $\mathrm{P}$ is the propulsive power output generated by the biathlete, $\mathrm{g}$ is the gravitational acceleration (i.e., 9.81 m/s^2^), *α* denotes the course inclination, and ${\upmu}_{\mathrm{r}}$ = 0.026 is the rolling friction coefficient of the roller ski wheels, measured using a towing test on a roller-ski treadmill, as previously described by Sandbakk et al. (2010). Furthermore, *ρ* denotes air density, ${\mathrm{C}}_{\mathrm{d}}$ is the effective drag coefficient, and $\mathrm{A}$ is the projected frontal area of the system.

Environmental wind was omitted from the model, since accurate consideration of wind effect along the complete courses is highly challenging, if not impossible (for the effect of environmental wind on power output against air drag, see Sundström et al. (2013)). Air density was calculated using environmental factors, including average air pressure and temperature throughout the trials, assuming dry air as:

2 $$\uprho =\overline{\mathrm{p} }/({\mathrm{R}}_{\mathrm{s}\mathrm{p}\mathrm{e}\mathrm{c}}\overline{\mathrm{T} }),$$

where $\overline{\mathrm{p} }$ is the average air pressure, $\overline{\mathrm{T} }$ is the average air temperature in Kelvin, and ${\mathrm{R}}_{\mathrm{s}\mathrm{p}\mathrm{e}\mathrm{c}}$ is the specific gas constant for dry air.

For the NR condition, ${\mathrm{m}}_{\mathrm{s}\mathrm{y}\mathrm{s}}$ was set to the mass of the participant, whereas under the WR condition it equaled the combined mass of the biathlete and the rifle.

In the power-balanced model, ${\mathrm{C}}_{\mathrm{d}}\mathrm{A}$ was treated as a single parameter and described as a function of sub-technique and speed, i.e., ${\mathrm{C}}_{\mathrm{d}}\mathrm{A}(\mathrm{S},\mathrm{v})$. This model for ${\mathrm{C}}_{\mathrm{d}}\mathrm{A}$ estimation was based on wind-tunnel measurements of Nordic combined athletes’ ${\mathrm{C}}_{\mathrm{d}}\mathrm{A}$ under dynamic conditions (Elfmark et al. 2023). Further methodological details are provided in the Supplementary material.

Based on Eq. (1), total external work against external resistive and inertial forces ($\mathrm{W}$) was calculated as:

3 $${\mathrm{W}}_{i}=\left({\mathrm{m}}_{\mathrm{s}\mathrm{y}\mathrm{s}}\left(v\frac{{\mathrm{dv}}_{i}}{{\mathrm{ds}}_{i}}+\mathrm{g}\left(\mathrm{sin}{\upalpha}_{i}+{\upmu}_{\mathrm{r}}\mathrm{cos}{\upalpha}_{i}\right)\right)+0.5\uprho {\mathrm{C}}_{\mathrm{d}}\mathrm{A}\left(\mathrm{S}, {\mathrm{v}}_{i}\right) {{\mathrm{v}}_{i}}^{2}\right){\mathrm{ds}}_{i},$$

where *i* denotes the *i*th meter of the respective course. In the tucked position (i.e., when $\mathrm{S}=0$), ${\mathrm{W}}_{i}$ was assumed to equal zero. External work performed over a given section of the course was defined as the sum of the performed work per meter within the section boundaries. External work performed during each completed lap, as well as over the entire trial, was calculated similarly.

Furthermore, the average propulsive power output for the *i*th course section, characterized by the corresponding absolute distance from the start position ${\mathrm{a}}_{\mathrm{j}}$ and section distance ${\mathrm{s}}_{\mathrm{j}}$, was calculated using Eq. (3) as:

4 $${\mathrm{P}}_{\mathrm{j}}=\frac{{\sum}_{\mathrm{i}={\mathrm{a}}_{\mathrm{j}}}^{{\mathrm{a}}_{\mathrm{j}}+{\mathrm{s}}_{\mathrm{j}}} {\mathrm{W}}_{\mathrm{i}}}{{\mathrm{t}}_{{\mathrm{a}}_{\mathrm{j}}+{\mathrm{s}}_{\mathrm{j}} }- {\mathrm{t}}_{{\mathrm{a}}_{\mathrm{j}}}},$$

where the denominator represents the section duration. Analogous to the calculation of performed external work, average propulsive power output was also calculated for each completed lap and the entire trial.

### Statistical analysis

All statistical analyses were performed using Jamovi (Jamovi, version 2.6.13, jamovi.org). Normality of all variables was assessed using the Shapiro–Wilk test, and most of the physiological variables were normally distributed. Variables were analyzed section by section as well as across the entire lap. For normally distributed variables, a 2 × 3 repeated measures Analysis of variance (ANOVA) (2 conditions WR/NR × 3 laps) was used. Assumption of sphericity was tested using Mauchly´s test, and if the assumption of sphericity was violated, the Greenhouse–Geisser correction was used. Physiological variables and time during the simulated shooting were analyzed using a 2 × 2 repeated measures ANOVA (2 conditions WR/NR × 2 simulated shootings). In cases of significant main or interaction effects, Bonferroni-adjusted post hoc tests were conducted to identify specific differences. Effect sizes were calculated as partial eta-squared (η^2^_p_) for all ANOVA interactions and interpreted as small (0.01), medium (0.06) or large (0.14) according to Cohen (1988). To compare the entire trial between conditions (WR *vs.* NR), a paired t-test was used, with effect size calculated using Cohen’s *d* and interpreted as small (0.2), medium (0.5) and large (0.8) effect.

For non-normally distributed data (time and speed for course sections, RPE and BF for sections and laps, body-mass normalized external work for laps), non-parametric tests were used. Differences between conditions (WR *vs.* NR) were assessed using the Wilcoxon signed-rank test, while differences across laps were examined using Friedman’s test. In case of a significant result in the Friedman’s test, a Durbin-Conover comparison was used to detect specific differences between laps. Data are presented as mean ± standard deviation (SD) except for RPE, which is presented as median (interquartile range (IQR)). The level of significance was set at α ≤ 0.05.

## Results

### Performance

Total race duration was 42.6 s longer for WR compared to NR (1194.5 ± 89.7 *vs.* 1151.9 ± 88.1 s, p < 0.001, *d* = 2.35), corresponding to an overall speed difference of 0.20 m∙s^−1^ (5.49 ± 0.47 *vs.* 5.69 ± 0.49 m∙s^−1^). The time differences between skiing WR and NR across laps 1‒3 were 13.5, 12.2 and 16.8 s, respectively, corresponding to average speed differences of 0.20, 0.18 and 0.25 m∙s^−1^. All lap-wise comparisons of time and speed showed statistically significant differences between conditions (all p < 0.05; Table 2). The main effect of condition in the ANOVA showed that skiing time in the WR condition was longer than in the NR condition for all sections (p < 0.05; Table 3). However, when investigating section-specific differences in time and speed between WR and NR, statistically significant differences were observed only for the uphill (all laps) and flat sections (lap 3) (Table 3, Fig. 4).

**Table 2:** Time, speed, physiological variables, power output, and external work rate for the three laps when skiing with a rifle (WR) and without a rifle (NR)

	Lap 1		Lap 2		Lap 3		ANOVA
	WR	NR	WR	NR	WR	NR	F-value, *p-value* (ES)
Time (s)	399.8 ± 29.8	386.3 ± 29.5^*^	399.9 ± 31.4	387.7 ± 31.0^*,3^	394.8 ± 30.5	378.0 ± 30.2*,^2^	^A^F_(1,19)_ = 114.5, < *0.001* (0.86), ^B^F_(2,38)_ = 9.5, *0.002* (0.33), ^C^F_(2,38)_ = 0.8, *0.408* (0.04)
Mean Speed (m·s^−1^)	5.46 ± 0.49	5.66 ± 0.48^*^	5.46 ± 0.46	5.64 ± 0.49^*,3^	5.53 ± 0.50	5.78 ± 0.54^*,2^	^A^F_(1,19)_ = 110.7, < *0.001* (0.85), ^B^F_(2,38)_ = 9.6, *0.002* (0.34), ^C^F_(2,38)_ = 1.2, *0.311* (0.06)
Mean HR (% HR_max_)	83.9 ± 3.1^2,3^	83.7 ± 3.6^2,3^	87.8 ± 3.0^1,3^	87.8 ± 3.3^1,3^	89.7 ± 2.5^1,2^	89.3 ± 3.2^1,2^	^A^F_(1,19)_ = 0.3, *0.585* (0.02), ^B^F_(2,38)_ = 214.7, < *0.001* (0.92), ^C^F_(2,38)_ = 0.7, *0.469* (0.03)
Mean HR (beats·min^−1^)	168 ± 6^2,3^	167 ± 8^2,3^	175 ± 7^1,3^	175 ± 7^1,3^	179 ± 8^1,2^	179 ± 8^1,2^	^A^F_(1,19)_ = 0.2, *0.658* (0.01), ^B^F_(2,38)_ = 188.3, < *0.001* (0.91), ^C^F_(2,38)_ = 0.4, *0.697* (0.02)
Mean VO_2_ (mL·kg^−1^·min^−1^)	48.1 ± 7.0^2^	47.8 ± 6.8^2^	49.8 ± 7.1^1^	49.1 ± 6.4^1^	49.2 ± 7.7	49.1 ± 6.4	^A^F_(1,15)_ = 0.0, *0.986* (0.00), ^B^F_(2,30)_ = 9.7, *0.004* (0.39), ^C^F_(2,30)_ = 0.1, *0.794* (0.01)
VO_2peak_ (mL·kg^−1^·min^−1^)	58.1 ± 8.8	57.5 ± 7.7	58.5 ± 8.7	57.4 ± 7.7	56.7 ± 8.3	56.8 ± 7.6	^A^F_(1,15)_ = 0.0, *0.919* (0.00), ^B^F_(2,30)_ = 3.0, *0.067* (0.17), ^C^F_(2,30)_ = 0.7, *0.504* (0.05)
Mean RER	0.98 ± 0.04	0.99 ± 0.04	0.97 ± 0.03	0.97 ± 0.03	0.98 ± 0.04	0.98 ± 0.03	^A^F_(1,15)_ = 0.1, *0.825* (0.00), ^B^F_(2,30)_ = 3.5, *0.043* (0.19), ^C^F_(2,30)_ = 1.1, *0.351* (0.07)
Mean VE (L·min^−1^)	119.7 ± 27.2^2,3^	119.7 ± 26.4^2,3^	126.8 ± 29.2^1^	127.9 ± 28.2^1,3^	131.8 ± 33.2^1^	133.6 ± 31.5^1,2^	^A^F_(1,19)_ = 0.4, *0.521* (0.02), ^B^F_(2,38)_ = 31.2, < *0.001* (0.62), ^C^F_(2,38)_ = 0.6, *0.462* (0.03)
Mean BF (breaths·min^−1^)	55.5 ± 6.0^2,3^	55.3 ± 5.2^2,3^	58.0 ± 5.4^1,3^	57.7 ± 4.4^1,3^	61.1 ± 5.3^1,2^	60.6 ± 4.7^1,2^	
Blood lactate (mmol·L^−1^)	7.78 ± 2.15^3^	7.37 ± 2.15^3^	8.08 ± 2.29^3^	7.83 ± 2.53^3^	9.36 ± 2.57^1,2^	9.40 ± 3.09^2,3^	^A^F_(1,19)_ = 0.7, *0.398* (0.04), ^B^F_(2,38)_ = 22.6, < *0.001* (0.54), ^C^F_(2,38)_ = 0.9, *0.405* (0.04)
RPE	16 (15–17)^2,3^	16 (15–17)^2,3^	18 (17–19)^1,3^	17 (16–18)^*,1,3^	19 (18–20)^2,3^	19 (18–20)^2,3^	
Mean power output (W)	171 ± 33	174 ± 35	170 ± 36	171 ± 35^3^	179 ± 41	183 ± 39^2^	^A^F_(1,17)_ = 5.2, *0.036* (0.23), ^B^F_(2,34)_ = 11.9, < *0.001* (0.41), ^C^F_(2,34)_ = 0.5, *0.625* (0.03)
Mean power output (W·kg^−1^)	2.42 ± 0.23	2.46 ± 0.23	2.40 ± 0.27^3^	2.41 ± 0.23^3^	2.52 ± 0.28^2^	2.59 ± 0.27^2^	^A^F_(1,17)_ = 5.0, *0.038* (0.23), ^B^F_(2,34)_ = 14.9, < *0.001* (0.47), ^C^F_(2,34)_ = 0.6, *0.541* (0.04)
External work rate (kJ)	69 ± 16	67 ± 16	68 ± 16	66 ± 16^3^	71 ± 18	70 ± 17^2^	^A^F_(1,17)_ = 10.8, *0.004* (0.39), ^B^F_(2,34)_ = 9.3, < *0.001* (0.35), ^C^F_(2,34)_ = 0.1, *0.920* (0.01)
External work rate (kJ·kg^−1^)	0.97 ± 0.12	0.95 ± 0.12^*^	0.95 ± 0.13^3^	0.93 ± 0.12^*,3^	0.99 ± 0.14^2^	0.98 ± 0.14^2^	

When the data were non-normally distributed, no ANOVAs was performed and only comparisons between conditions (WR and NR) and laps are presented

*HR*heart rate, *VO*_2_ oxygen uptake, *VO*_2peak_ peak oxygen uptake, *RER * respiratory exchange ratio, *VE* ventilation, *BF* breathing frequency, *RPE* ratings of perceived exertion, *ES* effect size

^*^Significantly different from WR within the same lap (p < 0.05): ^1,2,3^Significantly different from laps 1, 2, and 3, respectively, within the same condition (p < 0.05)

^A^Main effect of condition (WR vs. NR)

^B^Main effect of lap

^C^Interaction effect: condition × lap

**Table 3:** Time spent in uphill (U1, U2), flat (F1, F2, F3), and downhill (D1, D2) sections of the course while skiing with a rifle (WR) and without a rifle (NR), as well as the duration of the two simulated shootings (SS)

		Lap 1		Lap 2		Lap 3		ANOVA
	Section	WR	NR	WR	NR	WR	NR	F-value, *p-value* (ES)
Time (s)	F1	56.3 ± 7.0	55.0 ± 6.1	55.9 ± 5.9	54.3 ± 5.8	56.4 ± 6.5	53.7 ± 5.7^*^	^A^F_(1,19)_ = 11.4, *0.003* (0.38), ^B^F_(2,38)_ = 0.6, *0.557* (0.03), ^C^F_(2,38)_ = 1.4, *0.261* (0.07)
	U1	52.4 ± 8.1	49.9 ± 7.3	51.9 ± 7.5	49.7 ± 6.6^*^	52.3 ± 8.0	49.7 ± 6.8^*^	^A^F_(1,19)_ = 19.9, < *0.001* (0.51), ^B^F_(2,38)_ = 0.5, *0.546* (0.03), ^C^F_(2,38)_ = 0.1, *0.822* (0.01)
	D1	13.4 ± 1.1	12.9 ± 0.8	13.4 ± 0.9	13.0 ± 0.9	13.3 ± 1.0	13.3 ± 1.2	^A^F_(1,18)_ = 5.9, *0.026* (0.25), ^B^F_(2,36)_ = 1.1, *0.354* (0.06), ^C^F_(2,36)_ = 2.8, *0.76* (0.13)
	U2	110.3 ± 22.2	104.6 ± 21.0^*^	111.4 ± 22.5	105.5 ± 20.9^*^	111.5 ± 23.2	104.8 ± 20.9^*^	
	F2	14.9 ± 5.6	14.5 ± 5.4^*^	14.8 ± 5.5	14.5 ± 5.2	15.2 ± 5.5	14.4 ± 5.3^*^	
	D2	81.6 ± 19.7	80.2 ± 19.6^*^	81.9 ± 20.4	80.7 ± 19.7	82.0 ± 20.0	80.5 ± 19.9^*^	
	F3	69.3 ± 5.9^3^	67.4 ± 5.7^3^	68.5 ± 5.5^3^	68.0 ± 7.2^3^	62.0 ± 6.5^1,2^	59.7 ± 7.3^1,2^	^A^F_(1,19)_ = 14.4, *0.001* (0.43), ^B^F_(2,38)_ = 70.6, < *0.001* (0.79), ^C^F_(2,38)_ = 1.6, *0.216* (0.08)
	SS	41.5 ± 8.2	38.6 ± 7.9	36.2 ± 6.7	39.2 ± 9.4	-	-	^A^F_(1,19)_ = 0.0, *0.987* (0.00), ^B^F_(1,19)_ = 2.6, *0.127* (0.12), ^C^F_(1,19)_ = 5.1, *0.036* (0.21)

When the data were non-normally distributed, no ANOVAs was performed and only comparisons between conditions (WR and NR) and laps are presented

*ES* effect size

^*^Significantly different from WR within the same lap (p < 0.05): ^1,2,3^Significantly different from laps 1, 2, and 3, respectively, within the same condition (p < 0.05)

^A^Main effect of condition (WR vs. NR)

^B^Main effect of lap

^C^Interaction effect: condition × lap

### Physiological variables during roller-skiing

**Fig. 3:**
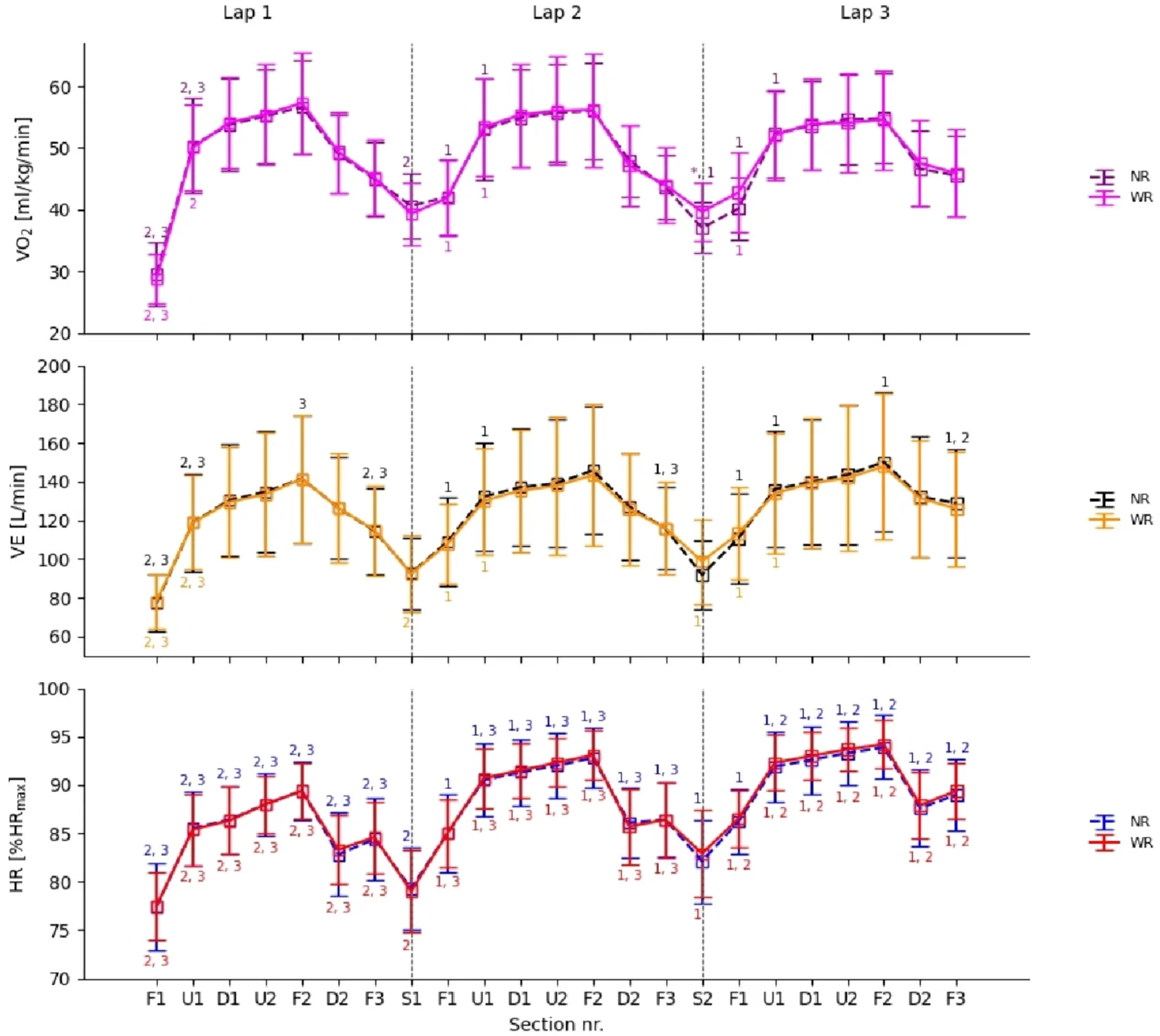
Mean oxygen uptake (VO_2_), ventilation rate (VE), and heart rate (HR) across course sections during roller-skiing with a rifle (WR) and without a rifle (NR), as well as during the two simulated shootings (S1 and S2). Whiskers indicate ± SD. * F* flat section, *U* uphill section, *D* downhill section; ^*^ = significantly different from WR within the same lap (p < 0.05): ^1,2,3^significantly different from laps 1, 2, and 3, respectively, within the same condition (p < 0.05)

There was no difference during roller-skiing between WR and NR for any of the physiological variables when comparing laps or specific sections (Tables 2 and 4, Fig. 3). However, there was a statistically significant difference between WR and NR in RPE for the second lap (Table 2). Mean HR, VE, BF and RPE increased across laps for both conditions, while BLa increased during the last lap compared to the first two laps for both WR and NR (Table 2).

**Table 4:** Respiratory exchange ratio (RER) and breathing frequency (BF) across uphill (U1, U2), flat (F1, F2, F3), and downhill (D1, D2) sections of the course while skiing with a rifle (WR) and without a rifle (NR), as well as the during the two simulated shootings (SS)

		Lap 1		Lap 2		Lap 3		ANOVA
	Section	WR	NR	WR	NR	WR	NR	F-value, *p-value* (ES)
Mean RER	F1	0.94 ± 0.07	0.94 ± 0.07	0.97 ± 0.03	0.96 ± 0.04	0.95 ± 0.04	0.96 ± 0.04	^A^F_(1,17)_ = 0.0, *0.849* (0.00), ^B^F_(2,34)_ = 2.5, *0.099* (0.13), ^C^F_(2,34)_ = 0.4, *0.681* (0.02)
	U1	0.93 ± 0.06	0.93 ± 0.05	0.92 ± 0.03	0.92 ± 0.03	0.92 ± 0.03	0.92 ± 0.03	^A^F_(1,17)_ = 0.3, *0.579* (0.02), ^B^F_(2,34)_ = 1.4, *0.253* (0.08), ^C^F_(2,34)_ = 0.4, *0.666* (0.02)
	D1	0.98 ± 0.06^3^	0.98 ± 0.06^3^	0.95 ± 0.04	0.95 ± 0.03	0.95 ± 0.03^1^	0.95 ± 0.03^1^	^A^F_(1,17)_ = 0.2, *0.685* (0.01), ^B^F_(2,34)_ = 10.8, *0.002* (0.39), ^C^F_(2,34)_ = 0.0, *0.960* (0.00)
	U2	1.00 ± 0.05	1.00 ± 0.05	0.98 ± 0.03	0.97 ± 0.03	0.98 ± 0.04	0.98 ± 0.04	^A^F_(1,17)_ = 0.6, *0.463* (0.03), ^B^F_(2,48)_ = 8.8, *0.003* (0.34), ^C^F_(2,34)_ = 0.5, *0.636* (0.03)
	F2	1.01 ± 0.05	1.01 ± 0.05	1.00 ± 0.03	0.99 ± 0.04	1.00 ± 0.05	1.00 ± 0.04	^A^F_(1,17)_ = 0.0, *0.850* (0.00), ^B^F_(2,34)_ = 2.4, *0.119* (0.13), ^C^F_(2,34)_ = 0.2, *0.738* (0.01)
	D2	1.03 ± 0.05	1.04 ± 0.04^2^	1.01 ± 0.05	1.02 ± 0.04^1^	1.02 ± 0.04	1.03 ± 0.05	^A^F_(1,15)_ = 1.2, *0.293* (0.07), ^B^F_(2,30)_ = 5.5, *0.009* (0.27), ^C^F_(2,30)_ = 0.0, *0.957* (0.00)
	F3	0.97 ± 0.04	0.97 ± 0.04	0.96 ± 0.04	0.95 ± 0.04	0.97 ± 0.05	0.97 ± 0.04	^A^F_(1,15)_ = 1.3, *0.277* (0.08), ^B^F_(2,30)_ = 4.4, *0.021* (0.23), ^C^F_(2,30)_ = 0.6, *0.577* (0.04)
	SS	0.94 ± 0.04	0.92 ± 0.04	0.93 ± 0.02	0.93 ± 0.03	-	-	^A^F_(1,17)_ = 3.7, *0.071* (0.18), ^B^F_(1,17)_ = 1.0, *0.328* (0.06), ^C^F_(1,17)_ = 3.9, *0.064* (0.19)
Mean BF (breaths min^-1^)	F1	55.6 ± 9.1	54.3 ± 7.2	55.8 ± 6.2^3^	56.4 ± 4.9^3^	58.1 ± 5.4^2^	58.8 ± 6.1^2^	
	U1	55.6 ± 8.7^3^	54.9 ± 8.1^2,3^	59.1 ± 6.3^3^	60.2 ± 5.8^1,3^	61.2 ± 6.2^1,2^	62.1 ± 6.1^1,2^	
	D1	55.1 ± 8.0^2,3^	55.6 ± 8.1^2,3^	58.4 ± 6.0^1^	59.8 ± 5.0^1^	60.8 ± 6.2^1^	60.4 ± 4.9^1^	
	U2	53.5 ± 6.2^2,3^	54.1 ± 5.0^3^	56.6 ± 6.1^1,3^	56.2 ± 5.2^3^	59.6 ± 7.3^1,2^	58.6 ± 6.2^1,2^	^A^F_(1,19)_ = 0.2, *0.643* (0.01), ^B^F_(2,38)_ = 25.8, < *0.001* (0.58), ^C^F_(2,38)_ = 1.8, *0.188* (0.09)
	F2	57.5 ± 5.5^3^	57.5 ± 6.0^2,3^	59.7 ± 5.7^3^	60.0 ± 4.9^1,3^	63.4 ± 7.2^1,2^	61.7 ± 5.4^1,2^	
	D2	56.7 ± 5.4^3^	55.8 ± 5.2^3^	59.2 ± 6.0	57.3 ± 4.8^3^	61.8 ± 5.6^1^	60.6 ± 5.5^1,2^	^A^F_(1,19)_ = 6.1, *0.023* (0.24), ^B^F_(2,38)_ = 34.0, < *0.001* (0.64), ^C^F_(2,38)_ = 0.7, *0.522* (0.03)
	F3	57.0 ± 6.2^3^	57.2 ± 5.7^3^	59.1 ± 6.2^3^	58.3 ± 6.6^3^	63.8 ± 6.8^1,2^	63.7 ± 4.8^1,2^	^A^F_(1,19)_ = 0.1, *0.784* (0.00), ^B^F_(2,38)_ = 26.5, < *0.001* (0.58), ^C^F_(2,38)_ = 0.2, *0.707* (0.01)
	SS	46.5 ± 5.0^2^	45.2 ± 4.0^2^	51.5 ± 6.0^1^	47.9 ± 4.1^*,1^	-	-	^A^F_(1,19)_ = 5.4, *0.032* (0.22), ^B^F_(1,19)_ = 47.8, < *0.001* (0.72), ^C^F_(1,19)_ = 4.3, *0.051* (0.19)

When the data were non-normally distributed, no ANOVAs was performed and only comparisons between conditions (WR and NR) and laps are presented

*ES* effect size

^*^Significantly different from WR within the same lap (p < 0.05): ^1,2,3^Significantly different from laps 1, 2, and 3, respectively, within the same condition (p < 0.05)

^A^Main effect of condition (WR vs. NR)

^B^Main effect of lap

^C^Interaction effect: condition × lap

### Physiological variables during simulated shooting

During the second simulated shooting, VO_2_ and BF were higher in the WR condition than in the NR condition (Table 4, Fig. 3), without significant differences in duration between conditions (Table 3). For both conditions, mean HR and RPE increased from the first to the second simulated shooting, whereas VE increased only in the WR condition (Table 4, Fig. 3).

### Power output and work rate during roller skiing

**Fig. 4:**
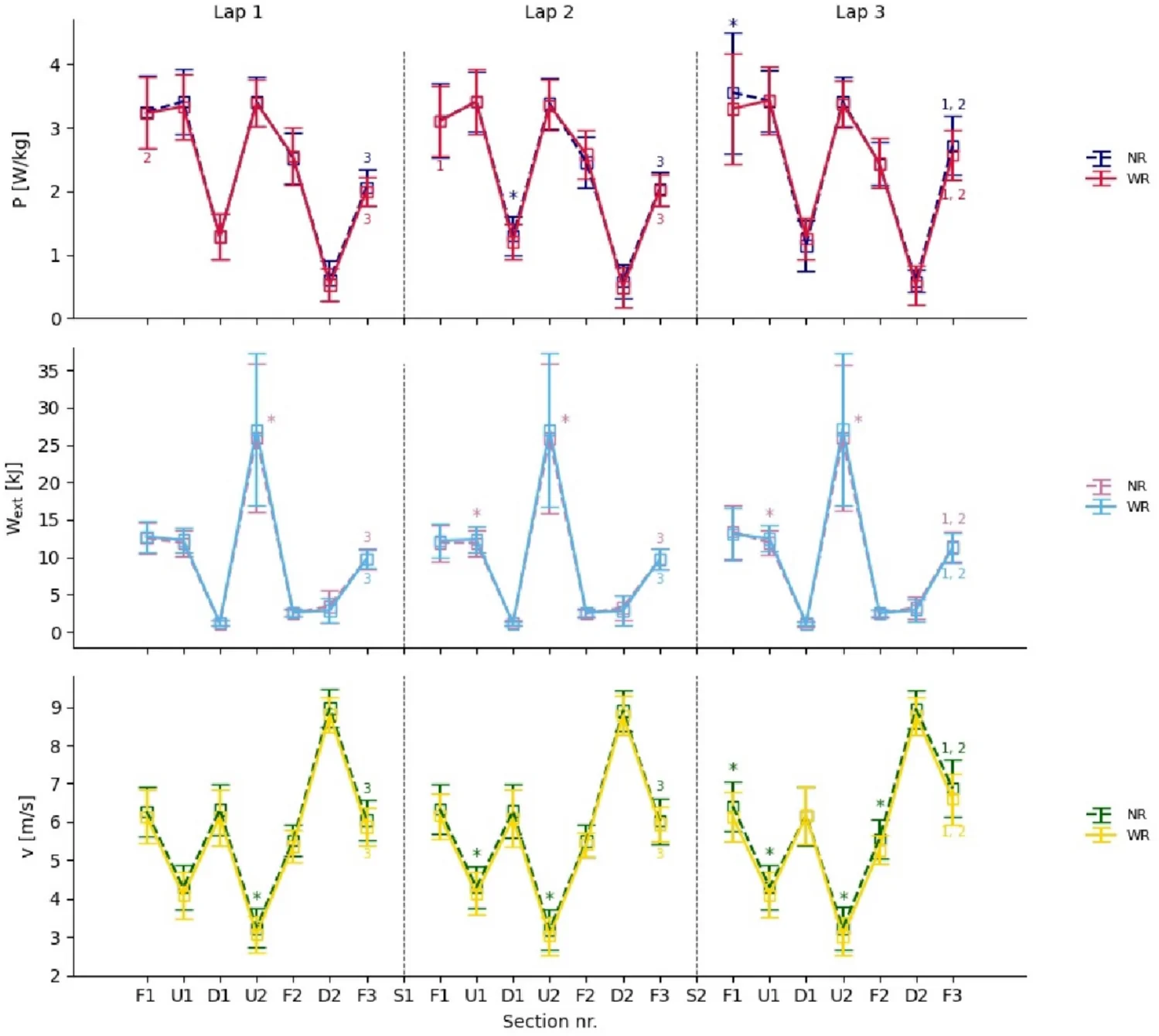
Mean body-mass normalized power output (P), external work (W_ext_), and speed (v) across course sections during roller-skiing with a rifle (WR) and without a rifle (NR). No values are presented for the two simulated shootings (S1 and S2) as the participants were standing still during these phases. Whiskers indicate ± SD. *F* Fat section; *U*  uphill section; *D * downhill section; ^*^significantly different from WR within the same lap (p < 0.05): ^1,2,3^significantly different from laps 1, 2, and 3, respectively, within the same condition (p < 0.05)

The participants produced a slightly lower average power output throughout their trial in the WR compared to the NR trial (absolute, 173 ± 36 *vs.* 176 ± 35 W, p = 0.040, *d* = 0.53; body-mass normalized, 2.45 ± 0.24 *vs.* 2.48 ± 0.22 W·kg^−1^, p = 0.043, *d* = 0.52). Contrarily, rifle carriage had a positive effect on the accumulated external work, with higher values observed in the WR condition (absolute, 207 ± 50 *vs*. 203 ± 49 kJ, p = 0.004, *d* = -0.78; body-mass normalized, 2.91 ± 0.37 *vs.* 2.85 ± 0.37 kJ·kg^−1^; p = 0.006, *d* = -0.72). Downhill sections were not completely without propulsion in any of the trials, and body-mass normalized power output was lower in the WR condition compared with NR during section D1 in lap 2 and section F1 in lap 3 (p < 0.05), with no other significant between-condition differences (Fig. 4). External work was higher in the WR than the NR condition for five of the six uphill sections in total (p < 0.05), whereas no differences between conditions were observed for flat or downhill sections (Fig. 4).

## Discussion

This is the first study comprehensively investigating the effect of rifle carriage on physiological responses as well as estimated power output and external work during a simulated biathlon roller-skiing sprint. The main results of this study were the following: 1) biathletes skied ~ 3.6% slower WR compared to NR, with the largest differences attained uphill; 2) physiological responses for roller-skiing WR did not differ from NR; 3) VO_2_, BF and RPE were higher during the second simulated shooting in the WR condition than in the NR condition; 4) power output were similar for skiing WR and NR, while 5) external work was greater for skiing WR compared to NR due to the longer duration of the WR trial; and 6) lap-to-lap pacing was more even in the WR condition, while participants demonstrated a considerably higher increase in speed over the last lap in the NR condition.

### Performance

One previous field study with skiing on snow has compared performance between WR and NR, showing a 4.3% difference in skiing time during one lap of skiing (~ 2200 m) (Jonsson Kårström et al. 2022), which closely aligns with the 3.6% performance gap observed in the present roller-skiing study. The difference in performance between WR and NR has been shown to be larger in a laboratory setting, with a ~ 6.5% performance decrement (Jonsson Kårström et al. 2019). However, the laboratory protocol solely consisted of uphill skiing in the Gear 3 sub-technique, where rifle carriage is expected to have the largest effect on performance. Contrarily, the present study included varied terrains and allowed free sub-technique selection.

Importantly, the magnitude of performance decrement associated with rifle carriage on skiing performance is fundamentally dependent on the ratio of the rifle mass and the biathlete’s body mass. This suggests a typically larger effect for female biathletes compared with the males, given similar rifle masses but typically lower body mass among the former. Supporting this notion, previous research has demonstrated that the time difference between skiing WR and NR in uphill sections was larger for female than for male biathletes (~ 15 *vs.* 4 s, respectively) (Jonsson Kårström et al. 2022). Although the investigation of sex differences was beyond the scope of the present study, such aspects should be addressed in future research.

### Physiological responses

There were no differences in physiological responses for the WR and NR roller-skiing trials, which is in line with findings from 3‒4 min treadmill roller-skiing time trials (Jonsson Kårström et al. 2019). In the study by Jonsson Kårström et al. (2019), WR and NR roller-skiing resulted in similar values for VO_2max_, aerobic energy contribution, maximal VE, peak HR, and BLa, which aligns with the presented field-based results. The physiological data from the present study also indicates that biathletes were able to utilize their aerobic and anaerobic capacities to the same degree during WR and NR skiing, which is supported by the similar values for mean VO_2_ and BLa between the two conditions. Also, HR for roller-skiing during both trials was in line with previous research demonstrating a mean HR of ~ 90% of HR_max_ during the second half of the race (Hoffman and Street 1992). Overall trends of continuously collected physiological responses revealed a gradually increasing HR throughout the trials, irrespective of condition (Fig. 3). In contrast, VO_2_ was relatively stable across laps, especially throughout sections U1‒F2, while VE demonstrated only a modest increase compared with HR. This is in line with the findings of Gløersen et al. (2024), which showed that mean VO_2_ was not affected by race duration in XC skiing. Notably, race duration int the present study was relatively short compared to a regular biathlon sprint competition, but still slightly longer than in the 1.6‒6.4 km trials in the study by Gløersen et al. (2024).

During the second simulated shooting, VO_2_, BF, and RPE were significantly higher for the WR than the NR condition. This may be explained by the slightly higher speed and power output (p > 0.05) during section F3 for WR compared with NR in the second lap, relative to the first lap. The mean difference in section duration for F3 between conditions was 2.1 and 2.3 s for the first and last laps, respectively, whereas the mean difference for lap 2 was only 0.5 s, further indicating a pacing-induced effect concerning the observed differences in physiological variables during the second simulated shooting. The delay in respiratory variables in relation to the onset of exercise (Born et al. 2017), may explain the differences in VO_2_ and BF during the simulated shooting, as a result of the relatively higher roller-skiing speed prior to the shooting range. This is an important factor to consider, since higher physiological load may negatively alter shooting performance, especially in the standing shooting position. Previous research has shown that shooting accuracy in the standing position decreases with higher HR (Danielsen et al. 2025; Hoffman et al. 1992), likely due to a decreased stability of hold, aiming accuracy, and cleanness of triggering (Ihalainen et al. 2018). Although no between-condition (i.e., WR *vs.* NR) differences in HR were found, the elevated VO_2_, BF, and RPE in the WR condition suggest a higher physiological and perceived load, which may nevertheless influence shooting outcomes. Luchsinger et al. (2019) showed that higher performing male biathletes decreased their skiing speed more during the final course section (~ 450 m) preceding the standing shooting than their lower-performing counterparts, which might form a viable strategy to increase the chance of a better shooting performance, and therefore overall biathlon performance. However, it is important to remember that no actual shooting was performed in this study, and even though the participants were instructed to pace themselves prior to the shooting range as if they were supposed to shoot, adherence to this instruction may have varied between individuals, posing a limitation to our result.

### Power output, external work rate and pacing

The first two laps were similar in duration for both WR and NR roller-skiing; however, biathletes were able to increase roller-skiing speed to a significantly greater extent during the final lap of NR skiing. Compared to NR, WR resulted in greater external work, particularly on uphill sections, primarily due to an increased section duration, as power outputs were similar for most of the sections. The resulting difference in accumulated external work throughout the first and second laps may have contributed to the smaller increase in skiing speed during the last lap for WR skiing, which raises the importance of a low fatigability as a factor for success in biathlon competitions (Meixner et al. 2025). Although the difference in accumulated external work between WR and NR trials can be considered relatively minor (4.2% greater external work in the WR condition), its potential impact should not be overlooked (Sánchez-Jiménez et al. 2026), especially when extrapolating these findings to biathlon races with longer durations, such as the individual competition. The simulated competition in the present study was relatively short (~ 17‒20 min), while the longest biathlon competitions comprise up to 40 min of skiing (Laaksonen et al. 2018b). Such a duration increase amplifies the accumulated work difference relative to NR skiing and potentially also the performance-deteriorating effects of skiing WR.

In alignment with previous findings in sprint and distance XC skiing (Gløersen et al. 2018b; Swarén and Eriksson 2019), biathletes demonstrated highly intermittent pacing strategies from a power-output perspective, irrespective of trial condition (WR or NR). The used power output estimation approach provided reasonable results, as power output across uphill sections averaged ~ 3.4 ± 0.8 W·kg^−1^ over a cumulative duration of 7‒9 min. Comparable values have been reported in a laboratory study, where biathletes of similar performance level to the participants in the present study, produced mean power outputs of 3.2‒4.4 W·kg^−1^ throughout a treadmill time-trial test at moderate inclines for ~ 3.5 min (Jonsson Kårström et al. 2019). Additionally, Stöggl et al. (2015) characterized elite-level biathletes’ competition load as 3.3‒3.7 W·kg^−1^ for moderate and steep inclines, respectively. Furthermore, estimated VO_2_ demands during uphill sections, calculated according to the approach described by Gløersen et al. (2018b), revealed that values exceeded the VO_2peak_ by a mean of 9% (range across all individual uphill sections: 1‒40%). Skiing condition had a moderate effect on mean power outputs throughout WR and NR trials, which may be partially attributed to the fact that the used ${\mathrm{C}}_{\mathrm{d}}\mathrm{A}$ model did not consider the aerodynamic effects of rifle carriage (see the Supplementary material).

Mean time differences between WR and NR trials averaged about 43 s, out of which, on average ~ 26 s (i.e., 61%) were accumulated uphill. During WR skiing, lap times were relatively consistent, demonstrating a 5.1 s difference between the slowest (lap 2) and fastest lap (lap 3) on a mean level. For NR skiing, the corresponding difference was larger, with a 9.7 s difference between the slowest lap (lap 2) and fastest lap (lap 3), meaning that participants demonstrated a considerably higher increase in speed over the last lap in the NR condition than the WR condition. Previous research has shown that XC skiers normally apply a positive pacing pattern, while biathletes demonstrate a more J-shaped lap-to-lap pacing pattern (Björklund and Laaksonen 2022; Formenti et al. 2015; Losnegard et al. 2016; Luchsinger et al. 2018; Stöggl et al. 2018). However, lap-to-lap durations in the present study did not show a clear J-shaped pacing pattern. Since NR in the present study showed a faster last lap (compared to the normal positive pacing pattern in XC skiing), the biathletes seem to have adopted a more “biathlon-like” pacing strategy (speed profile) across laps, potentially to facilitate shooting performance according to the given instructions.

### Methodological considerations

This is the first study to investigate the effects of rifle carriage on performance, physiological responses, power output and external work in a competition-like setup. However, there are some limitations of the study that are important to consider before interpreting the results in practice. First of all, a limitation to the presented results is that sprint competitions typically cover longer distances (i.e., 7.5 km for women and 10 km for men) than course distances in the present study. The course distance was shortened to limit the possible effect of fatigue from the first to the second trial. However, the skiing duration was similar to that during biathlon sprints at the highest competition level (Laaksonen et al. 2018b). One of the great challenges posed to field data collections is the variability of weather conditions. During the data collection, the temperature varied between different days of testing (11‒24 °C). However, the weather was stable during the tests for each participant and should therefore have a minimal impact on the results since the participants served as their own control in the chosen study design. Both WR and NR trials were performed within two hours to minimize the effect of weather conditions on the results. The active rest-period between trials was 30 min, which has been shown to be a sufficient recovery period in previous research (Staunton et al. 2022). In addition, the similar average power outputs between WR and NR trials (mean difference of 2.7 W) together with the absence of a difference in pre-start BLa (2.46 ± 0.85 *vs.* 2.23 ± 0.77 mmol·L^−1^, p > 0.05) in the present study indicate that the 30 min recovery period between the two simulated sprint races (~ 17‒20 min of high-intensity exercise interspersed with two ~ 40-s passive recovery periods) was sufficient for well-trained biathletes. In addition, the biathletes were conditioned to perform such a test protocol during training at this time of year.

The data collection was performed as roller-skiing during the summer in contrast to biathlon competitions which are held on snow. There were two main reasons for this choice. Firstly, the gas analyzer that was used in this study is very sensitive to low temperatures. During pilot testing, we have observed that the sampling line freezes quickly at temperatures around and below 0 °C, leading to ice blockages. Secondly, on-snow skiing involves greater variability in conditions, including differences in temperature, snow type, snow preparation, and ski-waxing. In contrast, roller-skiing allows all athletes to use the same pair of roller skis, which improves the comparability of the trials. Together, these factors were considered decisive for conducting the tests using roller skiing instead of on-snow skiing. However, previous research has identified biomechanical differences between roller-skiing and on-snow skiing (Myklebust et al. 2022), which should be considered when interpreting the results.

In the present study, no real shooting was performed, which might have affected the pacing pattern of the participants. The used study design was chosen due to the placement of the breath-by-breath system on the chest and the mask, which prevented the participants from taking off their rifle and from accurate aiming. In addition, for four of the athletes, the data for gas exchange was not complete due to blockage of the sample line connected to the gas analyzers. Therefore, statistical analyses of VO_2_ and RER were only performed on 16‒19 participants, which may have affected the power of the analysis, although previous research on biathlon has shown that 10‒17 participants seem to be enough to detect physiological differences between roller-skiing WR and NR in laboratory settings (Jonsson Kårström et al. 2019; Rundell and Szmedra 1998; Stöggl et al. 2015).

Concerning the power output estimation method, additional assumptions not detailed under the Methods were included. Firstly, as evidenced by Eq. (1), the calculation of the frictional force did not account for centripetal forces due to the course’s curvature, in contrast to Gløersen et al. (2018b). Furthermore, the roller-skis were assumed to cover the same distance as the GNSS receiver and the shear forces, as well as the temperature and normal-force dependence of ${\upmu}_{\mathrm{r}}$ were neglected (Ainegren et al. 2008; Gløersen et al. 2018b). It is believed that the warm-up period provided enough time for the roller-ski wheel bearings to reach a stable operating temperature before the participant commenced their first trial. Similar to environmental wind, breaking forces (i.e., negative power output production) were left unconsidered; however, based on the given course profiles and the nature of roller-skiing, this rather introduced a minor error into the derived average power output values. For detailed insights onto the estimation of the air drag, the reader is referred to the Supplementary material.

### Practical applications

In the present study, respiratory variables were significantly higher for WR during the second (standing) simulated shooting compared to NR. However, the increased load on the respiratory system could be a result of an altered pacing pattern over the course sections approaching the shooting range. Since elevated physiological markers during shooting may have a negative effect on especially standing shooting outcomes, it is recommended that biathletes optimize their lap-by-lap pacing strategies for maximizing their performance. The result of this study also showed that the greatest difference in roller-skiing speed between WR and NR was during the last lap of a simulated competition, which may be attributed to the higher accumulated work performed during roller-skiing WR. This suggests the importance of a low fatigability specific to WR skiing in biathlon performance, and demands biathletes and coaches to prioritize WR skiing, especially during high-intensity training sessions, to develop endurance performance factors specific to biathlon.

## Conclusion

To our knowledge, this is the first study that has investigated the effects of rifle carriage on performance, physiological responses, power output and external work during a simulated biathlon competition in the field. The present study showed that although biathletes exhibit declined performance due to rifle carriage, they can produce similar power outputs relative to NR skiing during a simulated competition. Furthermore, it was demonstrated that the primary driving factor behind greater accumulated external work and metabolic energy expenditure during skiing WR is the elongated work duration. Importantly, no significant differences were found in physiological responses between skiing WR and NR, but the pacing strategy prior to the shooting range increased physiological responses during the second simulated shooting, which may affect shooting performance. Biathletes should apply optimized pacing strategies and consider their perception of effort and power output during competitions, especially when approaching the shooting range.

## Supplementary Information

Below is the link to the electronic supplementary material.

Supplementary file1 (DOCX 261 KB)

## Data Availability

The datasets generated and analyzed during the current study are available from the corresponding author on reasonable request.

## Abbreviations

ANOVA: Analysis of variance
BF: Breathing frequency
BLa: Blood lactate concentration
CO_2_: Carbon dioxide production
D: Downhill
ES: Effect size
F: Flat
GNSS: Global navigation satellite system
HR: Heart rate
HR_max_: Maximum heart rate
IBU: International Biathlon Union
IMU: Inertial measurement units
IQR: Interquartile range
NR: With no rifle
RER: Respiratory exchange ratio
RPE: Rating of perceived exertion
SD: Standard deviation
SS: Simulated shooting
U: Uphill
VE: Minute ventilation
VO_2_: Oxygen uptake
VO_2max_: Maximum oxygen uptake
VO_2peak_: Peak oxygen uptake
WR: With rifle
XC: Cross-country
