# Supplementary material for: Effects of rifle carriage on performance, physiological responses, power output and external work in a simulated biathlon competition
Source: Eur J Appl Physiol. 2026 Jun 29;126(10):5319–33. doi: 10.1007/s00421-026-06301-x (PMC13633986; doi:10.1007/s00421-026-06301-x)
Supplement: Supplementary file 1 [file 421_2026_6301_MOESM1_ESM.pdf]

Supplementary material

Extension on deriving reference trajectories

The participants' inertia was considered when determining the length of high-speed downhill sections (i.e., section D2 for both sexes), aiming to exclude the initial meters of low-gradient terrain from the subsequent flat sections where the participants were holding the tucked downhill position, and there was no active propulsion. These course segments could be well-identified based on altitude signals plotted against the accumulated distance over the courses. Since all the participants rose from the tucked position to start poling assisted propulsion at approximately the same point of the courses, this created an artificial "bump" in the altitude signal – which was not part of the real courses, nor removed by the GNSS receiver's proprietary filters. Assuming an average difference in the GNSS receiver's position relative to the ground of 1 meter between the tucked position and propulsion, these artificial inaccuracies were eliminated from the signals using a smoothing function.

Prediction of aerodynamic drag along the race courses

Aerodynamic drag (C_dA) plays an important role during cross-country skiing and biathlon, influencing the power required to maintain speed, as well as the maximum speed reached during downhill sections. Drag force is determined by the skier's frontal area, drag coefficient, air density, and the relative velocity of the skier with respect to the surrounding air (Ingen Schenau & Cavanagh, 1990). Frontal area can be estimated dynamically using inertial sensors on the torso and thighs, with segment orientations converted to pitch angles to predict effective cross-sectional area (Gløersen et al., 2018), or alternatively approximated using sigmoid functions and allometric scaling based on body mass (Moxnes et al., 2014; Sundström et al., 2013). The drag coefficient is dependent on the Reynolds number and can be modelled using logistic functions fitted to empirical data (Gløersen et al., 2018).

In the present study, C_dA was handled as a single parameter and predicted as a function of sub-technique selection and headwind speed relative to the (i.e., the skier's speed of travel, as environmental wind speed was assumed to be zero in all calculations) based on previously published empirical data. Linear regressions of the form $C_dA(v) = av + b$ were fitted to allometrically scaled values of C_dA measured for two Nordic combined athletes in a wind tunnel under dynamic conditions by Elfmark et al. (2023), thus adjusting them to each biathlete's body size (i.e., body mass) according to the scaling approach described by Sundström et al. (2013). The regression equations were fitted to the scaled C_dA values corresponding to three distinct headwind speed values per investigated sub-technique (i.e., Gears 3 and 4), resulting in coefficient of determination values of $R^2 = 0.84$ for the Gear 3, and $R^2 = 0.94$ for the Gear 4 sub-technique. For the tuck position, C_dA was modeled independent of speed by the allometric scaling of the reference value 0.27 m^2, reported by Spring et al. (1988) for a reference skier of 80 kg body mass, based on Sundström et al. (2013), nevertheless, propulsive power output was set to zero when the tuck position was detected along the courses. The values resulting from this process are presented in Figure A.1, as the mean predicted C_dA along the female and male courses.

Concerning the limitations of the applied approach, the aerodynamic effects of rifle carriage were not considered. Consequently, the relationships used to predict C_dA during the WR and NR trials were assumed to be

identical. However, in reality C_dA for a given sub-technique at a given speed is expected to be marginally higher for WR compared to NR skiing, including the tuck position. Additionally, the same relationship between C_dA and v were assumed for Gear 2 as for Gear 3. Nevertheless, this simplification - due to the lack of available data on aerodynamics in the Gear 2 sub-technique - is of minor importance, as skiers typically travel at their lowest speeds when employing the Gear 2 sub-technique (Andersson et al., 2010), resulting in a relatively small contribution of power against air drag to the total power output for this sub-technique (i.e., typically around 10-15% of the total power output). Furthermore, due to a lack of a sufficient number of (v; C_dA) coordinates, linearity was assumed between headwind speed relative to the skier and dynamic C_dA (i.e., air drag measured under dynamic conditions). This assumption is speculative rather than empirically established, particularly for the Gear 3 sub-technique, which exhibited root mean square errors of approximately 5% relative to the mean predicted C_dA values. Contrarily, the same metric was valued around 1% for Gear 4 models. Although previous research has demonstrated an effect of cycle rate (i.e., movement frequency) on the dynamic C_dA, this factor was omitted from the present C_dA model, as its influence was considered marginal (Elfmark et al., 2023). Future studies should explore these relationships through wind-tunnel testing across different sub-techniques and evaluate the aerodynamic effects of rifle carriage on power output to improve the accuracy of C_dA predictions within ecologically valid experimental settings in cross-country skiing and biathlon.

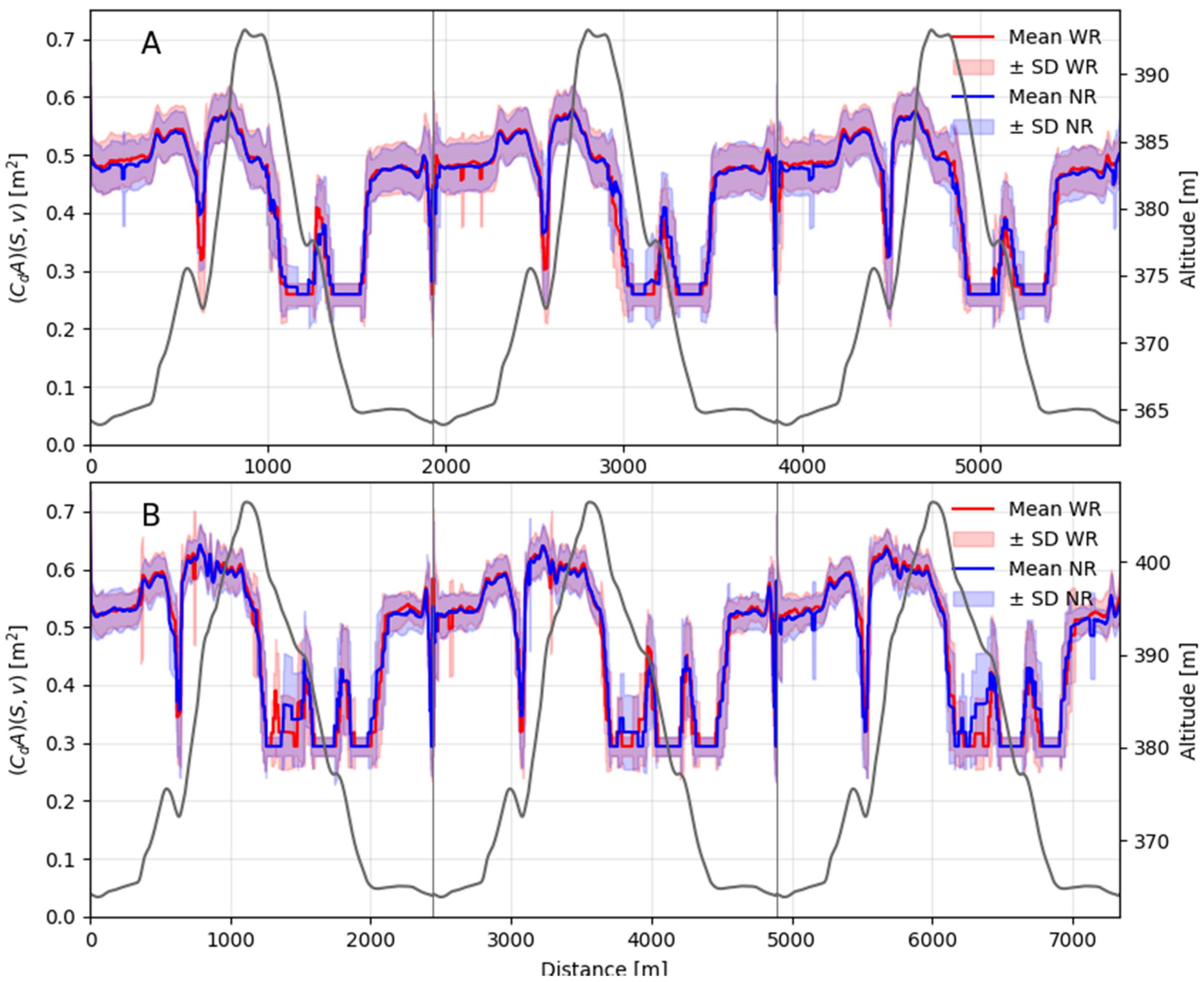

Fig. A.1 Aerodynamic drag (C_dA) estimated as a function of sub-technique (S) and speed (v) along the female course (panel A) and male course (panel B) during skiing with a rifle (WR; solid red line) and without a rifle (NR; solid blue line). Data are presented as means for ten female and eight male participants, respectively. Shaded areas indicate ± SD.

References

ANDERSSON, E., SUPEJ, M., SANDBAKK, Ø., SPERLICH, B., STÖGGL, T. & HOLMBERG, H. C. 2010. Analysis of sprint cross-country skiing using a differential global navigation satellite system. *Eur J Appl Physiol,* 110**,** 585-595.

ELFMARK, O., DOCTER, H., SANDBAKK, Ø. & KOCBACH, J. 2023. The influence of sub-technique and skiing velocity on air drag in skating style cross-country skiing. *Sports Engineering,* 26**,** 37.

GLØERSEN, Ø., LOSNEGARD, T., MALTHE-SØRENSSEN, A., DYSTHE, D. K. & GILGIEN, M. 2018. Propulsive Power in Cross-Country Skiing: Application and Limitations of a Novel Wearable Sensor-Based Method During Roller Skiing. *Frontiers in Physiology,* Volume 9 - 2018.

INGEN SCHENAU, G. J. V. & CAVANAGH, P. R. 1990. Power equations in endurance sports. *Journal of Biomechanics,* 23**,** 865-881.

MOXNES, J. F., SANDBAKK, Ø. & HAUSKEN, K. 2014. Using the power balance model to simulate cross-country skiing on varying terrain. *Open Access Journal of Sports Medicine,* 5**,** 89-98.

SPRING, E., SAVOLAINEN, S., ERKKILÄ, J., HÄMÄLÄINEN, T. & PIHKALA, P. 1988. Drag Area of a Cross-Country Skier. *International Journal of Sport Biomechanics,* 4**,** 103-113.

SUNDSTRÖM, D., CARLSSON, P., STÅHL, F. & TINNSTEN, M. 2013. Numerical optimization of pacing strategy in cross-country skiing. *Structural and Multidisciplinary Optimization,* 47**,** 943-950.
